# Transcriptome analysis of PK-15 cells expressing CSFV NS4A

**DOI:** 10.1186/s12917-022-03533-9

**Published:** 2022-12-12

**Authors:** Huifang Lv, Zhifeng Peng, Bingxin Jia, Huiyuan Jing, Sufang Cao, Zhikun Xu, Wang Dong

**Affiliations:** Key Laboratory of Veterinary Biological Products, College of Veterinary Medicine, Henan University of Animal Husbandry and Economy, 450046 Zhengzhou, China

**Keywords:** RNA-seq, Classical swine fever virus, NS4A, Virus replication, Immune defence

## Abstract

**Background:**

Classical swine fever (CSF) is a severe disease of pigs that results in huge economic losses worldwide and is caused by classical swine fever virus (CSFV). CSFV nonstructural protein 4 A (NS4A) plays a crucial role in infectious CSFV particle formation. However, the function of NS4A during CSFV infection is not well understood.

**Results:**

In this study, we used RNA-seq to investigate the functional role of CSFV NS4A in PK-15 cells. A total of 3893 differentially expressed genes (DEGs) were identified in PK-15 cells expressing NS4A compared to cells expressing the empty vector (NC). Twelve DEGs were selected and further verified by RT‒qPCR. GO and KEGG enrichment analyses revealed that these DEGs were associated with multiple biological functions, including cell adhesion, apoptosis, host defence response, the inflammatory response, the immune response, and autophagy. Interestingly, some genes associated with host immune defence and inflammatory response were downregulated, and some genes associated with host apoptosis and autophagy were upregulated.

**Conclusion:**

CSFV NS4A inhibits the innate immune response, and suppresses the expression of important genes associated with defence response to viruses and inflammatory response, and regulates cell adhesion, apoptosis and autophagy.

**Supplementary Information:**

The online version contains supplementary material available at 10.1186/s12917-022-03533-9.

## Background

Classical swine fever virus (CSFV) is a causative pathogen of classical swine fever (CSF), one of the most economically disastrous diseases in the swine industry [1, 2]. The major symptoms of CSF include hyperthermia and systemic bleeding. Host cell defence mechanisms are essential for resistance to viral infection, such as the innate immune response and apoptosis. However, the innate immune response is inhibited by CSFV due to weak induction of antiviral cytokines, including type I interferon (IFN), and proinflammatory cytokines, such as IFN-β and interleukin (IL)-6 [3–5]. In addition, CSFV does not induce cell death [6, 7]. CSFV infection triggers a complete autophagic response to promote virus replication [8].

CSFV belongs to the *Flaviviridae* family and the *Pestivirus* genus [9]. It is a single-stranded positive-sense RNA virus. The CSFV genome is approximately 12.3 kb in length and encodes a polyprotein precursor that is further proteolytically processed into four structural proteins (C, E^rns^, E1 and E2) and eight nonstructural proteins (N^pro^, p7, NS2, NS3, NS4A, NS4B, NS5A and NS5B) [10, 11]. CSFV NS4A is an 8 kDa protein that is located in the nucleus and cytoplasm [12]. NS4A is a cofactor of the NS3 protease that assists the cleavage of downstream nonstructural proteins and is involved in the formation of infectious virions. CSFV NS4A induces IL-8 production by enhancing the mitochondrial antiviral signalling protein (MAVS) pathway and promoting CSFV replication [12]. However, the function of NS4A during CSFV infection needs to be further elucidated.

RNA-seq is a powerful approach to quantify and identify differentially expressed genes on a genome-wide scale and has been employed to study various viral infections and viral proteins [13, 14]. The aim of the current study was to use the RNA-seq method to investigate the functional role of CSFV NS4A in PK-15 cells. The results may provide novel information that will increase our understanding of the roles of NS4A in the pathogenesis of CSFV.

## Results

### Construction of PK-15 cells expressing NS4A

**Fig. 1:**
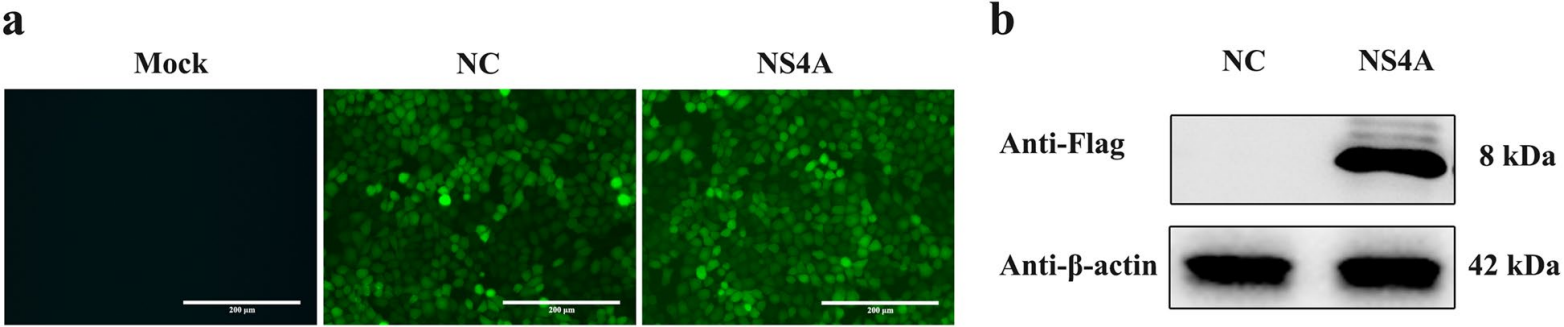
Confirmation of PK-15 cells expressing NS4A. **a** Confirmation of PK-15 cells expressing NS4A by fluorescence detection of the GFP reporter. Mock represents PK-15 cells; NC represents PK-15 cells expressing the empty vector; NS4A represents PK-15 cells expressing NS4A. **b** Western blotting for NS4A expression in PK-15 cells expressing NS4A. β-Actin was used as an internal control. The blots were cropped from the original blot by using Adobe Photoshop CS6, and the full-length original blots and multiple exposure images are included in additional files 3, 4 and 5

To analyse the function of CSFV NS4A, we first constructed PK-15 cells expressing NS4A by lentiviral infection. PK-15 cells were infected with lentiviral products overexpressing NS4A and screened with puromycin. Subsequently, the cells were examined via fluorescence detection of the GFP reporter and western blotting with a Flag antibody. As shown in Fig. 1a, green fluorescence in PK-15 cells expressing the empty vector (NC) or PK-15 cells expressing NS4A (NS4A) was visible under an inverted fluorescence microscope, and the percentage of positively infected cells was ~ 100%. However, no green fluorescence was visible in noninfected PK-15 cells. Additionally, NS4A expression was confirmed by western blot analysis using an anti-Flag mAb in NS4A cells (Fig. 1b), indicating that PK-15 cells expressing CSFV NS4A were successfully constructed.

### RNA sequencing and read assembly

In this study, sequencing libraries were prepared in triplicate, and RNA-seq was used to compare the gene expression levels of PK-15 cells expressing NS4A with those of PK-15 cells expressing the empty vector. RNA-seq generated 287,527,934 raw reads, with an average of 47,921,322 reads per sample. After ribosomal RNA and low-quality reads were discarded, a total of 272,843,899 clean reads were generated, with an average of 45,473,983 reads per sample. On average, 94.90% of the sequences was mapped onto the *Sus scrofa* reference genome (GCF_000003025.6_Sscrofa11.1). Among the mapped reads, 91.12% were mapped to a single position (Table 1).

**Table 1:** Statistics of the RNA-seq datasets

Sample	Raw reads	Clean reads	Total mapped reads	Uniquely mapped reads	Q30
NS4A_1	46,460,452	44,559,659	95.91%	91.86%	95.11%
NS4A_2	46,880,086	43,608,804	93.02%	89.52%	95.14%
NS4A_3	50,249,962	46,873,463	93.28%	89.73%	95.09%
NC_1	48,041,194	45,999,470	95.75%	91.94%	95.06%
NC_2	49,467,120	47,385,340	95.79%	91.93%	95.20%
NC_3	46,429,120	44,417,163	95.67%	91.75%	95.16%

### Differentially expressed genes (DEGs) in PK-15 cells expressing CSFV NS4A

**Fig. 2:**
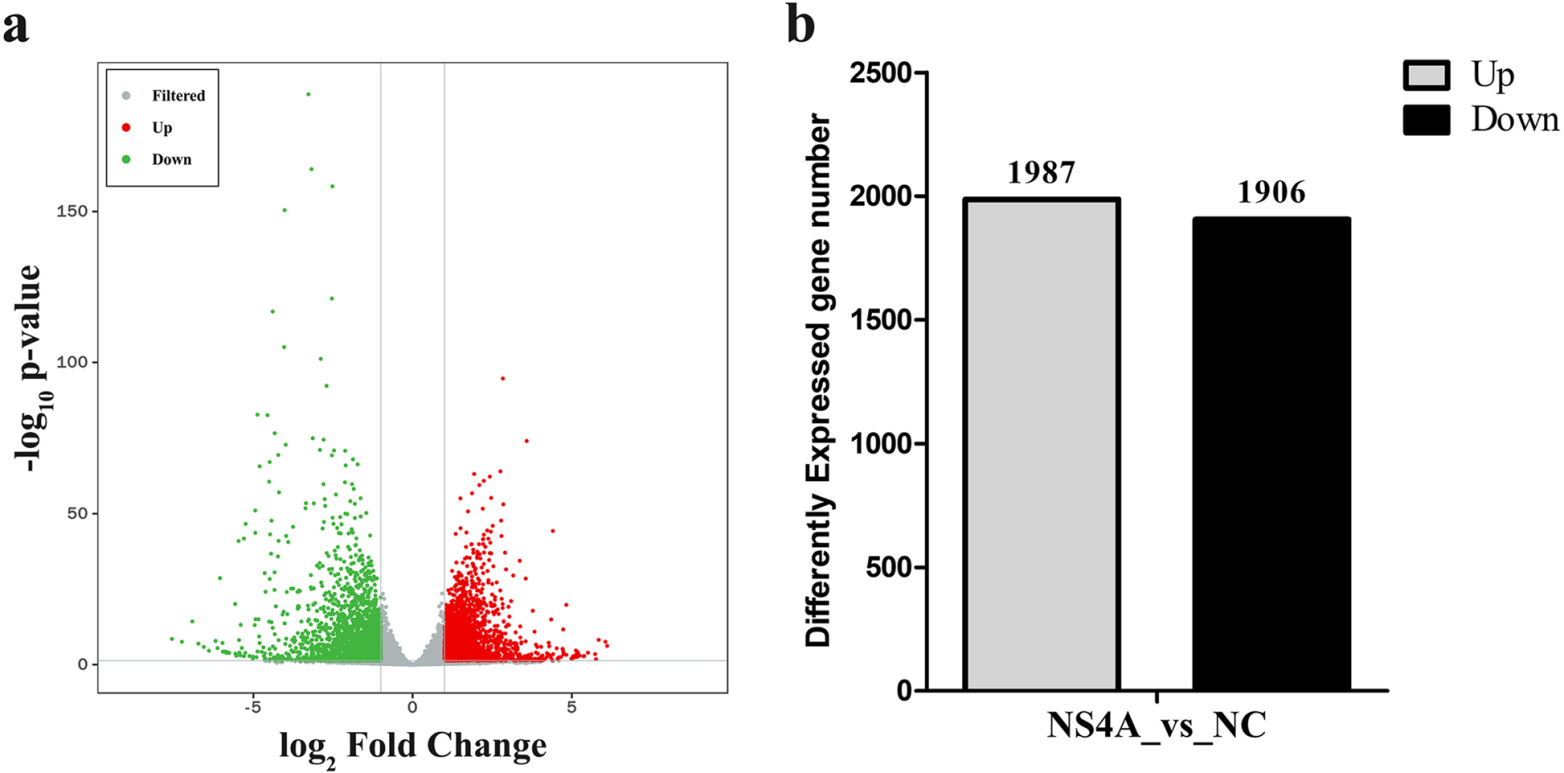
Statistical analysis of the DEGs. **a** Volcano plot displaying the DEGs in PK-15 cells expressing NS4A. In the figure, the two vertical grey lines represent the threshold of 2 times differential expression, and the horizontal grey line is the threshold of a *P *value = 0.05. Red dots indicate groups of upregulated genes, green dots indicate groups of downregulated genes, and grey dots indicate non-significantly differentially expressed genes. **b** The number of upregulated genes and downregulated genes

**Fig. 3:**
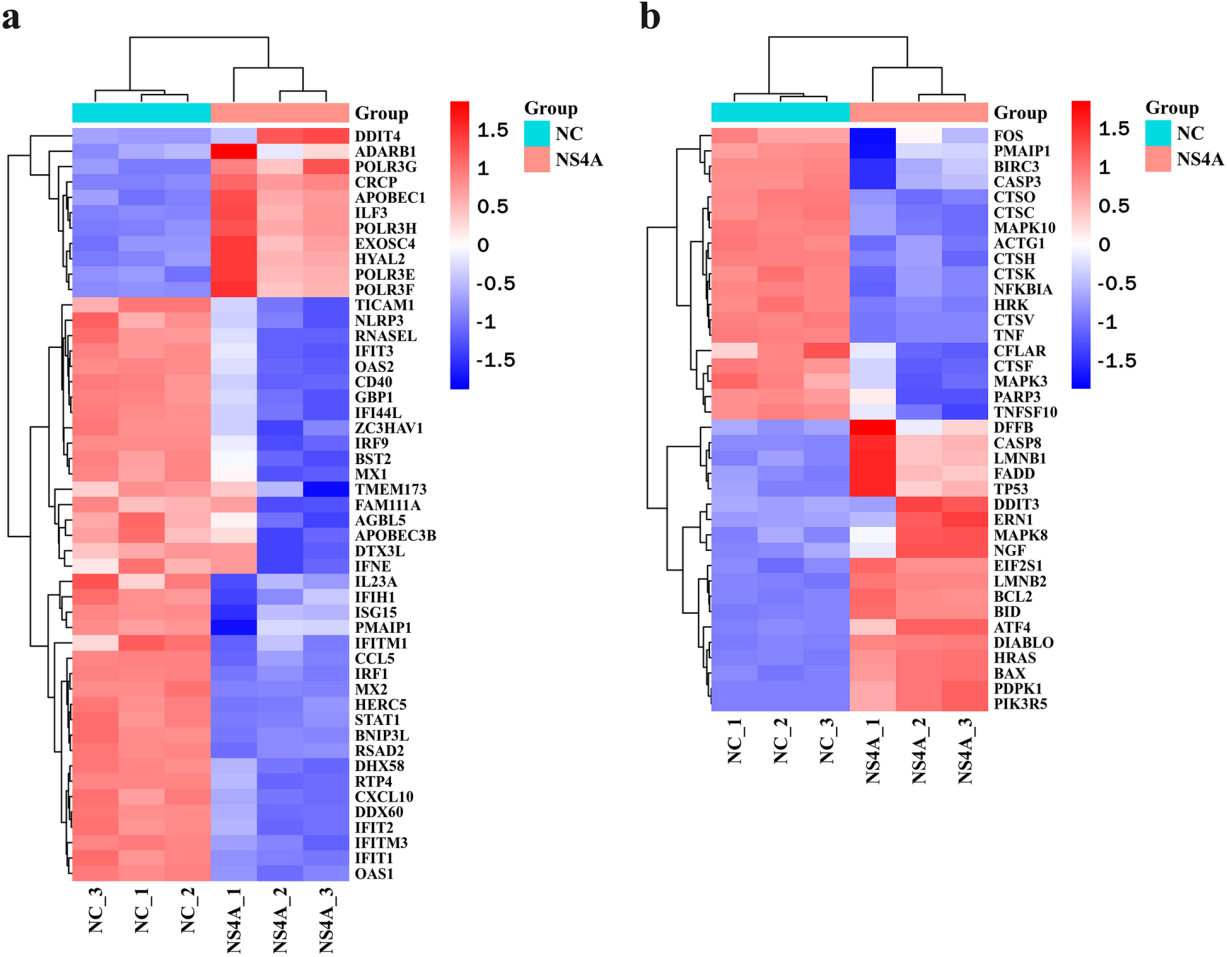
Expression profiles (heatmaps) of viral defence response genes and apoptosis genes in PK-15 cells expressing NS4A. The samples NC_1, NC_2, NC_3 and NS4A_1, NS4A_2, NS4A_3 are similar, but three independent experiments were performed. Each column is a sample, and each row represents the expression level of the same gene in different samples. The colour in the heatmap represents the gene expression change. Red indicates high gene expression, green indicates low gene expression, and white indicates unaltered gene expression. The DEGs are labelled on the right of the heatmap. **a** Heatmap of viral defence response genes. **b** Heatmap of apoptosis genes in response to viral infection

To compare the DEGs in the NC PK-15 cells with those in the PK-15 cells expressing NS4A, we identified DEGs with volcano plots (Fig. 2a). In all, 3893 genes were identified as DEGs in PK-15 cells expressing NS4A compared to NC PK-15 cells; 1987 DEGs were upregulated, and 1906 were downregulated (Fig. 2b). Additional file 1 shows the DEGs that were upregulated and downregulated. The most upregulated gene in PK-15 cells expressing NS4A was found to be mucin 12 gene (MUC12), which increased 68.95 fold change. The most downregulated gene in PK-15 cells expressing NS4A was found to be IL-10, which reduced 188.61 fold change. In addition, important immune response genes were downregulated, including TLR2, TRIF and STAT1 genes associated with TLR pathway, NOD1, NLRP3 and ERK genes associated with NLR pathway and RIG-I, MDA5, LGP2 and IFN-ε genes associated with RLR pathway. IL-1β, CXCL10, IL-10, and TNF-α genes, important inflammatory factors, were significantly downregulated. ITGB1/2/6/8, NECTIN2/3/4 and CLDN1/6/7/12/16, cell adhesion regulator genes, were significantly downregulated. However, other important cell adhesion genes were upregulated in PK-15 cells expressing NS4A, such as VEGF-A and VEGF-B. ATG3 genes associated with autophagy were upregulated. Furthermore, we screened 49 DEGs related to the defence response to viruses (Fig. 3a) and 38 DEGs related to apoptosis (Fig. 3b), and the DEGs were further analysed according to hierarchical clustering. Numerous genes associated with defence response to viruses were mainly some interferon stimulating genes (ISGs), and these genes were significantly downregulated, such as IFITM1/3, MX1, IFIT1, OAS1, BST-2 and ISG15. The significantly upregulated important genes related to apoptosis were FADD, Bid, Bax, Caspase 8, p53, ATF4, and DDIT3, whereas the Fos gene was significantly downregulated in PK-15 cells expressing NS4A compared to NC PK-15 cells. The analysis of RNA-seq data revealed that CSFV NS4A was involved in cell adhesion, cell apoptosis, host defence response, the inflammatory response, the immune response, and autophagy.

### Gene Ontology (GO) analyses and KEGG pathway analyses of DEGs

**Fig. 4:**
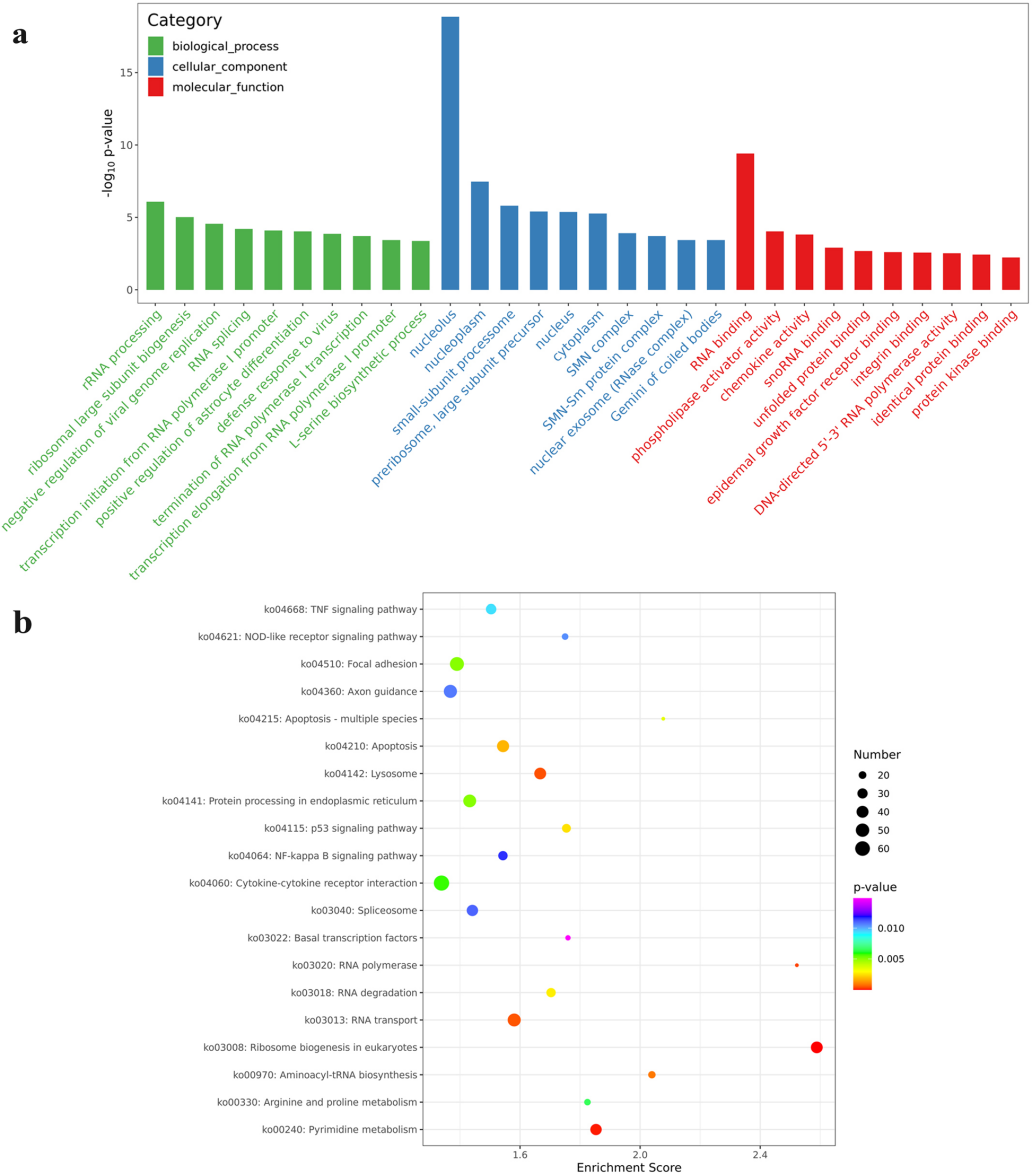
Functional enrichment analysis of DEGs expressed in PK-15 cells expressing NS4A. **a** Top 30 GO terms of the DEGs. The GO terms were classified into 3 categories, including BP, CC, and MF. The top 10 GO terms with the smallest *P* values or most significant enrichment in each GO category were selected. **b** Top 20 KEGG pathways of the DEGs. The top 20 pathways with the smallest *P* values or most significant enrichment were selected. The KEGG pathway database is obtained in KEGG software from the Kanehisa laboratory

To explore the biological functions of the DEGs, GO enrichment analysis was conducted. The DEGs were classified into three main categories: biological process (BP), cellular component (CC), and molecular function (MF). Additional file 2 shows that these DEGs were enriched with 4397 GO terms, including 637 GO terms in the CC category, 867 GO terms in the MF category, and 2893 terms in the BP category. According to *P < 0.05* as the standard, these DEGs were significantly enriched with 320 GO terms, and the top 30 GO terms are listed in Fig. 4a. The most significantly enriched term was nucleolus (GO: 0005634), followed by RNA binding (GO: 0003723), rRNA processing (GO: 0006364), and others. Many DEGs were enriched with the apoptotic process (GO: 0006915), defence response to virus (GO: 0051607) and negative regulation of viral genome replication (GO: 0045071) terms, such as Bid, DDIT3, TNF, ISG15, MX1, OAS1, BST2, IFIT1, and IFITM1.

KEGG pathway analysis is used for predicting the biological processes and phenotypic traits of genes. To analyse the function of CSFV NS4A, the DEGs were mapped to reference canonical signalling pathways via KEGG database analysis. According to a *P* value *<* 0.05 as the standard, the DEGs were significantly enriched in 30 pathways, and the top 20 enriched KEGG pathways are shown in Fig. 4b. The most significant pathway was cytokine‒cytokine receptor interaction (ko04060), in which 65 genes were differentially expressed. In addition, the apoptosis pathway (ko04210) and NOD-like receptor signalling pathway (ko04621) were found among these pathways, which are related to apoptosis and the immune system.

### Validation of RNA-seq data by real-time quantitative PCR (RT‒qPCR)

**Fig. 5:**
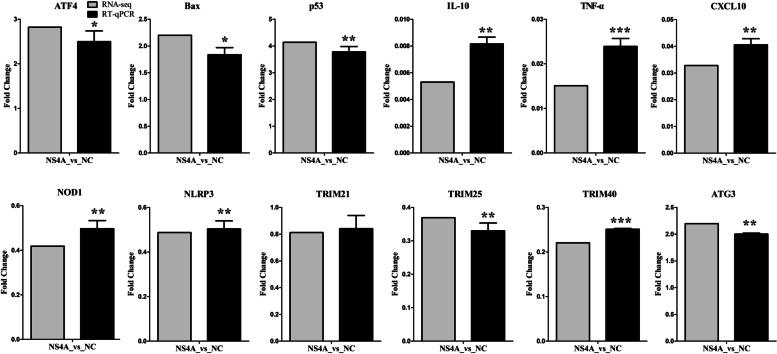
Validation of the RNA-seq data by RT‒qPCR. To confirm the results from RNA-seq, RT‒qPCR was performed using total RNA from NS4A- or NC-expressing PK-15 cells. β-Actin was used as a reference. The y-axis represents the normalized fold changes in the expression of these selected genes. The error bars represent the mean ± SD (*n* = 3). *, *P < 0.05*; **, *P < 0.01*; ***, *P < 0.001*

To validate the gene expression results obtained from RNA-seq, we tested the expression of important apoptosis-related genes (ATF4, Bax, p53), inflammatory response genes (IL-10, TNF-α, CXCL10), innate immune response genes (NOD1, NLRP3, TRIM21, TRIM25, TRIM40) and autophagy genes (ATG3) using RT‒qPCR. As shown in Fig. 5, the mRNA expression of ATF4, Bax, p53 and ATG3 were significantly upregulated, and the mRNA expression of IL-10, TNF-α, CXCL10, NOD1, NLRP3, TRIM25 and TRIM40 were significantly downregulated in NS4A-expressing cells. Comparison of the RNA-seq results and RT‒qPCR results of the selected genes showed that the two detection methods were largely consistent.

## Discussion

As a highly contagious and fatal viral disease, CSF has caused great economic losses in the swine industry worldwide [1, 2]. Many researchers have worked to reveal the pathogenesis of CSFV and have already made certain achievements. However, the functions of CSFV proteins need to be further studied. CSFV NS4A is a small nonstructural protein, and little is known about its function. In this study, RNA-seq was used to explore the effect of CSFV NS4A in PK-15 cells to analyse the function of NS4A during CSFV infection.

RNA-seq revealed 3893 DEGs in PK-15 cells expressing CSFV NS4A. These DEGs were related to cell adhesion, apoptosis, host defence response, the inflammatory response, the immune response, autophagy and other terms. Mucin 12 gene (MUC12) is a member of the mucin family and play an essential role in forming protective mucous barriers on epithelial surfaces. We observed that MUC12 were significantly upregulated in PK-15 cells expressing NS4A. CSFV infection causes diffuse haemorrhaging in the skin, kidneys, and other organs of pigs [15]. We found that VEGF-A and VEGF-B were upregulated in PK-15 cells expressing NS4A. VEGF is a multifunctional cytokine and leads to a powerfully increased vascular permeability [16], which may be closely related to haemorrhagic symptoms. In addition, cell adhesion molecules, such as integrin subunit beta 1/2/6/8 (ITGB1/2/6/8), nectin cell adhesion molecule 2/3/4 (NECTIN2/3/4) and the tight junction (TJ) protein claudin-1/6/7/12/16 (CLDN1/6/7/12/16), were significantly downregulated in NS4A-expressing cells; these molecules contribute to the maintenance and regulation of vascular permeability [17–19].

The virus replication process includes adsorption, internalization, uncoating, biosynthesis, assembly and release. A previous study has demonstrated that NS4A promotes CSFV replication [12]. However, the molecular mechanism has remained unclear. In this study, we observed that some important genes associated with defence response to viruses (IFITM1/3, MX1, IFIT1, OAS1, BST-2 and ISG15) were significantly downregulated in NS4A-expressing cells. Interferon-induced transmembrane protein 1/3 (IFITM1/3) inhibits the entry of viruses into the host cell cytoplasm and prevents subsequent viral fusion and release of viral contents into the cytosol. IFITM1/3 overexpression significantly inhibits CSFV replication [20]. Interferon-induced GTP-binding protein Mx1 (MX1) targets viruses through binding and inactivation of their ribonucleocapsids and inhibits CSFV replication [21]. Interferon-induced protein with tetratricopeptide repeats 1 (IFIT1) inhibits virus replication and translation initiation. Upregulation of the IFIT1 gene is closely linked to the replication inhibition of many viruses, including hepatitis E virus and hepatitis C virus (HCV) [22, 23]. 2’-5’-Oligoadenylate synthetase 1 (OAS1) activates latent RNase L, which results in viral RNA degradation and inhibition of virus replication and mediates the antiviral effect against HCV, severe acute respiratory syndrome coronavirus 2 (SARS-CoV-2), and dengue virus (DENV) [24–26]. Bone marrow stromal cell antigen 2 (BST-2) efficiently blocks the release of diverse mammalian enveloped viruses and represses the replication of HCV, Newcastle disease virus (NDV), vesicular stomatitis virus (VSV), and herpes simplex virus (HSV) [27]. Through covalent binding with the host and viral target proteins, interferon-stimulating gene 15 (ISG15) inhibits the release of viral particles and hinders virus replication. Previous studies have shown that the activity of ISG15 inhibits CSFV replication via inhibition of autophagy by ISGylating BECN1 [28]. The results indicate that CSFV NS4A might facilitate CSFV replication by reducing the expression of these antiviral genes.

In the early stages of virus infection, the innate immune system can be triggered to limit the spread of viruses. First, multiple pattern recognition receptors (PRRs) are activated by sensing of pathogen-associated molecular patterns (PAMPs), such as viral proteins and nucleic acids, leading to induction of cytokines and type I interferons. The intracellular PRRs mainly include Toll-like receptors (TLRs), RIG-I-like receptors (RLRs) and NOD-like receptors (NLRs) [29–32]. Previous studies have demonstrated that CSFV infection upregulates the expression of TLR2, TLR4 and TLR7 and downregulates the expression of TLR3 and TIR-domain-containing adapter-inducing interferon-β (TRIF) [5]. In addition, CSFV is recognized by RIG-I and MDA5 receptors to activate MAVS expression [3, 33]. CSFV infection can sufficiently activate assembly of the NLRP3 inflammasome [34]. In our study, downregulation of NLRs, such as NOD1, NLRP3, ERK and IL-1β, was observed. The TLR pathway was also inhibited, as evidenced by the downregulation of TLR2, TRIF, tumour necrosis factor-alpha (TNF-α) and STAT1. We further found that NS4A expression reduced the expression of RIG-I, MDA5, LGP2, IFN-ε and CXCL10, suggesting that the RLR pathway was also inhibited. TRIM proteins have emerged as important positive and negative regulators of PRR signalling pathways. Many TRIM proteins play central roles in the host defence against virus infection[35]. We observed that the expression of TRIM25 and TRIM40 were downregulated in NS4A-expressing cells. These results indicate that NS4A acts as a negative regulator of host innate defence and inhibits the innate immune response during CSFV infection, which might promote persistent CSFV infection.

Apoptosis plays an important role in preventing the virus from replicating and producing progeny viruses [36]. In vitro, CSFV infection does not induce apoptosis or pathological cell changes. Previous studies have shown that CSFV N^pro^, E^rns^ and NS2 proteins can inhibit apoptosis [7, 37, 38]. However, the effect of CSFV NS4A on apoptosis is unknown. Here, we observed that the expression of many apoptosis-associated genes was changed. The proto-oncogenes Fos and TNF-α, which have been associated with apoptotic cell death [39, 40], were downregulated in NS4A-expressing cells. In addition, FADD, Bid, Bax, Caspase 8, p53, ATF4, and CHOP, which are important apoptosis-associated genes [41–43], were upregulated in NS4A-expressing cells. Our results provide a reference for studying the relationship between NS4A and apoptosis.

Cells adapt to poor survival conditions through the initiation and execution of autophagy [44]. CSFV infection triggers a complete autophagic response to promote virus replication [8]. Autophagy-related 3 (ATG3), which is a ubiquitin-like-conjugating enzyme involved in autophagy [45], was upregulated in NS4A-expressing cells, implying that NS4A might be involved in the induction of autophagy during CSFV infection. Proinflammatory cytokines are involved in a wide variety of processes, stimulating the activation and migration of immune cells to infected sites and promoting virus clearance. Our results showed that the expression of IL-1β, CXCL10, IL-10, and TNF-α was reduced in NS4A-expressing cells. IL-1β and CXCL10, important inflammatory factors, are induced during CSFV infection [46, 47]. Additionally, TNF-α, a prototypical proinflammatory cytokine with pleiotropic activities, is secreted in CSFV-infected pigs and limits the replication of CSFV in cell culture [48]. These results indicate that CSFV NS4A might inhibit these important proinflammatory responses to promote virus replication, which is consistent with our previous results [12]. However, further studies are necessary to elucidate the mechanism of cytokine regulation in NS4A-expressing cells.

This finding indicates that CSFV NS4A might contribute to virus replication by inhibiting host cell protection and inducing host cell autophagy. Taken together, the findings of this study provide useful information for further understanding the function of CSFV NS4A during CSFV infection.

## Conclusion

In summary, our findings revealed 3893 DEGs in PK-15 cells expressing CSFV NS4A compared to NC cells via RNA-seq. These DEGs were involved in inflammatory responses, cell adhesion, apoptosis, host defence response, the immune response, autophagy and other processes. Interestingly, NS4A protein expression in PK-15 cells resulted in downregulation of genes involved in host immune defence and inflammatory response and upregulation of genes involved in host apoptosis and autophagy. Based on above results, this finding indicates that CSFV NS4A inhibits the innate immune response, and suppresses the expression of important genes associated with defence response to viruses and inflammatory response, and regulates cell adhesion, apoptosis and autophagy.

## Methods

### Cell culture and plasmid construction

HEK293T (ATCC, CRL-11,268) and PK-15 cells (ATCC, CCL-33) were preserved in our laboratory and cultured in high-glucose DMEM (GIBCO, UK) supplemented with 10% heat-inactivated foetal bovine serum (FBS) (HyClone, USA) at 37 °C and 5% CO_2_. The pCDH-CMV-NS4A-Flag vector encoding the CSFV NS4A protein with a Flag fusion protein at its C-terminus was constructed by cloning the NS4A gene into the overexpression lentivector pCDH-CMV-MCS-EF1-GreenPuro (CD513B-1) (SBI, Mountain View, CA, USA) using *Eco*R I and *Bam*H I restriction enzymes. The primers are listed in Supplementary Table S1, and the plasmid was confirmed by restriction digestion and sequencing.

### Construction of PK-15 cells expressing NS4A

HEK293T cells were cotransfected with the pCDH-CMV-NS4A-Flag plasmids and three other plasmids (pGag/Pol, pRev, pVSVG) using TurboFect (Thermo Fisher Scientific, USA). After 16 h, the medium was replaced with Advanced DMEM. The culture supernatants containing lentivirus were collected after 48 h. PK-15 cells were infected with lentiviral products expressing NS4A and then selected with 6 µg/mL puromycin (Thermo, USA) for 10 days. To confirm that PK-15 cells expressed NS4A, green fluorescence in PK-15 cells was confirmed to be visible under an inverted fluorescence microscope (Nikon, Japan). Then, NS4A gene expression was analysed by western blotting in PK-15 cells expressing NS4A. In brief, PK-15 cells expressing NS4A were harvested and lysed. Protein samples were separated by 12% SDS‒PAGE. According to the size of target proteins and the indication of protein marker, the unused parts of the separating gel and stacking gel were cut off. The proteins were transferred onto PVDF membranes (Millipore, USA). The membranes were incubated with an anti-β-actin mouse mAb (Tianjin Sungene Biotech, KM9001, China) or anti-Flag mouse mAb (CWBIO, China) at 4 °C overnight and then incubated with HRP-conjugated goat anti-mouse IgG (1:5000) (Bioss, China) for 2 h at room temperature. Then, the signal was detected using an automatic chemiluminescence image analysis system (Tanon, 5200, China).

### RNA isolation and library preparation

Total RNA was extracted using TRIzol reagent according to the manufacturer’s protocol. RNA purity and quantification were evaluated using a NanoDrop 2000 spectrophotometer (Thermo Scientific, USA). RNA integrity was assessed using the Agilent 2100 Bioanalyzer (Agilent Technologies, Santa Clara, CA, USA). Then, the libraries were constructed using a TruSeq Stranded mRNA LT Sample Prep Kit (Illumina, San Diego, CA, USA) according to the manufacturer’s instructions. Transcriptome sequencing and analysis were conducted by OE Biotech Co., Ltd. (Shanghai, China).

### RNA sequencing and differentially expressed gene analysis

The libraries were sequenced on an Illumina HiSeq X Ten platform, 150 bp paired-end reads were generated, and raw reads for each sample were generated. The raw data (raw reads) in FASTQ format were first processed using Trimmomatic [49], and the low-quality reads were removed to obtain clean reads. Then, the clean reads were mapped to the *Sus scrofa* genome (GCF_000003025.6_Sscrofa11.1) using HISAT2 [50]. The FPKM value [51] of each gene was calculated using Cufflinks [52], and the read counts of each gene were obtained by HTSeqcount [53]. Differential expression analysis was performed using the DESeq R package. A *P* value < 0.05 and fold change > 2 or fold change < 0.5 were set as the thresholds for significant differential expression.

### GO functional enrichment and pathway analysis

Hierarchical cluster analysis of the DEGs was performed to demonstrate the expression patterns of the genes in different groups and samples. GO enrichment and KEGG [54] pathway enrichment analyses of the DEGs were performed using R based on the hypergeometric distribution. The KEGG pathway database from the Kanehisa laboratory was used for KEGG pathway enrichment analyses [55–57].

### RT‒qPCR to validate gene expression

To validate the RNA sequencing results, 12 genes were selected and verified by RT‒qPCR with the specific primers in Supplementary Table S1. Total cellular RNA was extracted with TRIzol (Invitrogen, USA). Subsequently, the total RNA was firstly treated with DNase to remove genomic DNA, and cDNA was synthesized using a PrimeScript RT reagent kit (Vazyme, China). The relative mRNA expression was calculated with an ABI7500 system (Biosystem, CA, USA), with β-actin as the reference, using UltraSYBR Mixture (CWBIO, China) according to the manufacturer’s protocol. The data were analysed by using the 2^−∆∆Ct^ method. All experiments were performed in triplicate, and the results represent the mean ± standard deviation (SD).

### Statistical analysis

The RT‒qPCR experiments were performed at least three times, and the results represent the mean ± standard deviation (SD) of three replicates. The data were analysed by one-way ANOVA and Bonferroni post hoc test using SPSS software (version 18.0). A *P* value < 0.05 was considered to indicate significance.

## Supplementary Information

**Additional file 1:** **Supplementary Table S2.** Up-regulated and down-regulated genes in CSFV NS4A expressing PK-15 cells compared to those in NC expressing PK-15 cells.

**Additional file 2:** **Supplementary Table S3.** The GO terms of DEGs.

**Additional file 3.**

**Additional file 4.**

**Additional file 5.**

**Additional file 6:** **Supplementary Table S1.** Primers used in this study.

## Data Availability

All data generated or analysed during this study are included in this published article and its additional files, and the dataset analysed during the current study is available in the GEO repository at the NCBI under accession number GSE212691.
