# Supplementary figures and images for: Transcriptome analysis of PK-15 cells expressing CSFV NS4A
Source: BMC Vet Res. 2022 Dec 12;18:434. doi: 10.1186/s12917-022-03533-9 (PMC9742017; doi:10.1186/s12917-022-03533-9)

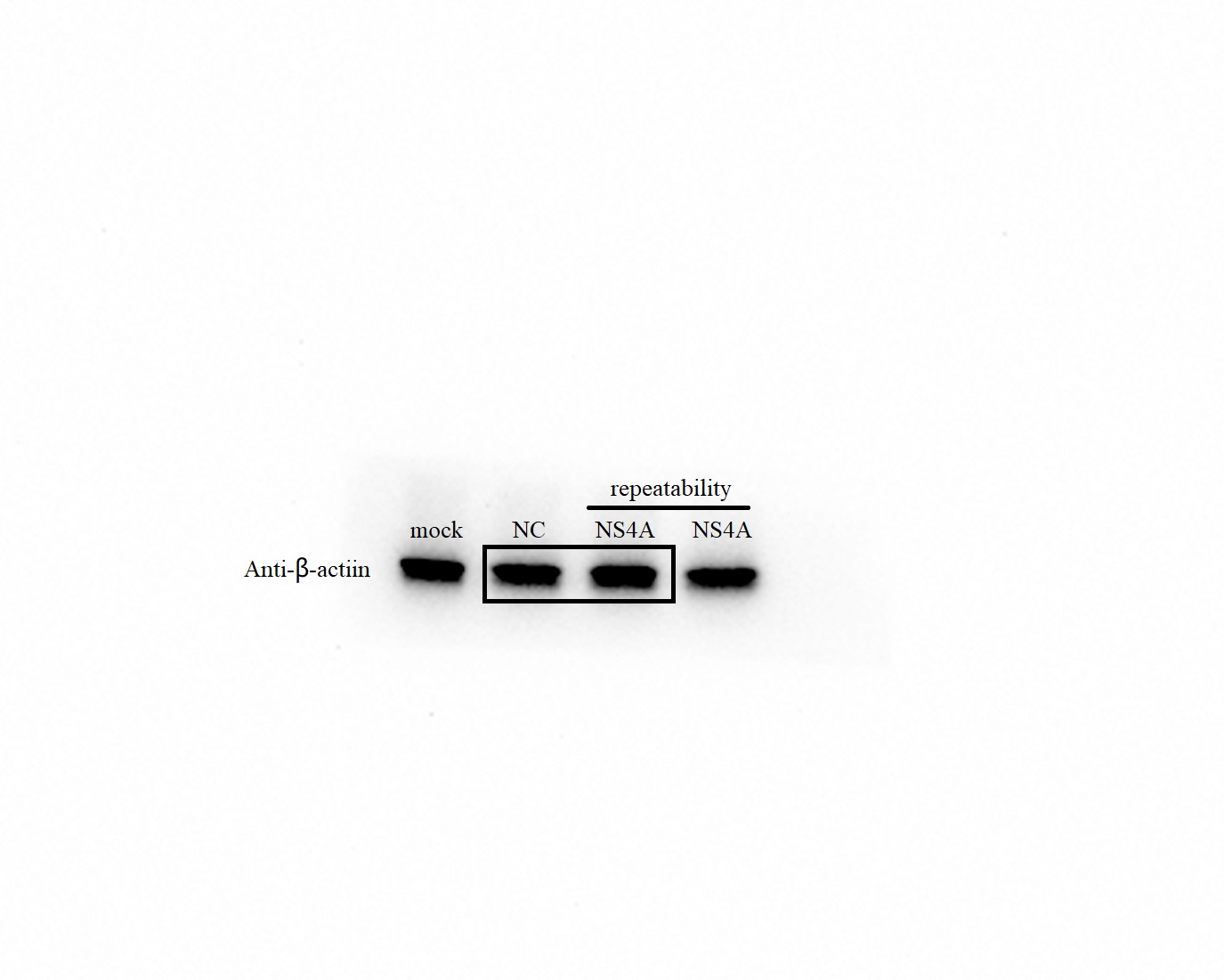

Supplement: Supplementary file 3 — Additional file 3. [file 12917_2022_3533_MOESM3_ESM.jpg]

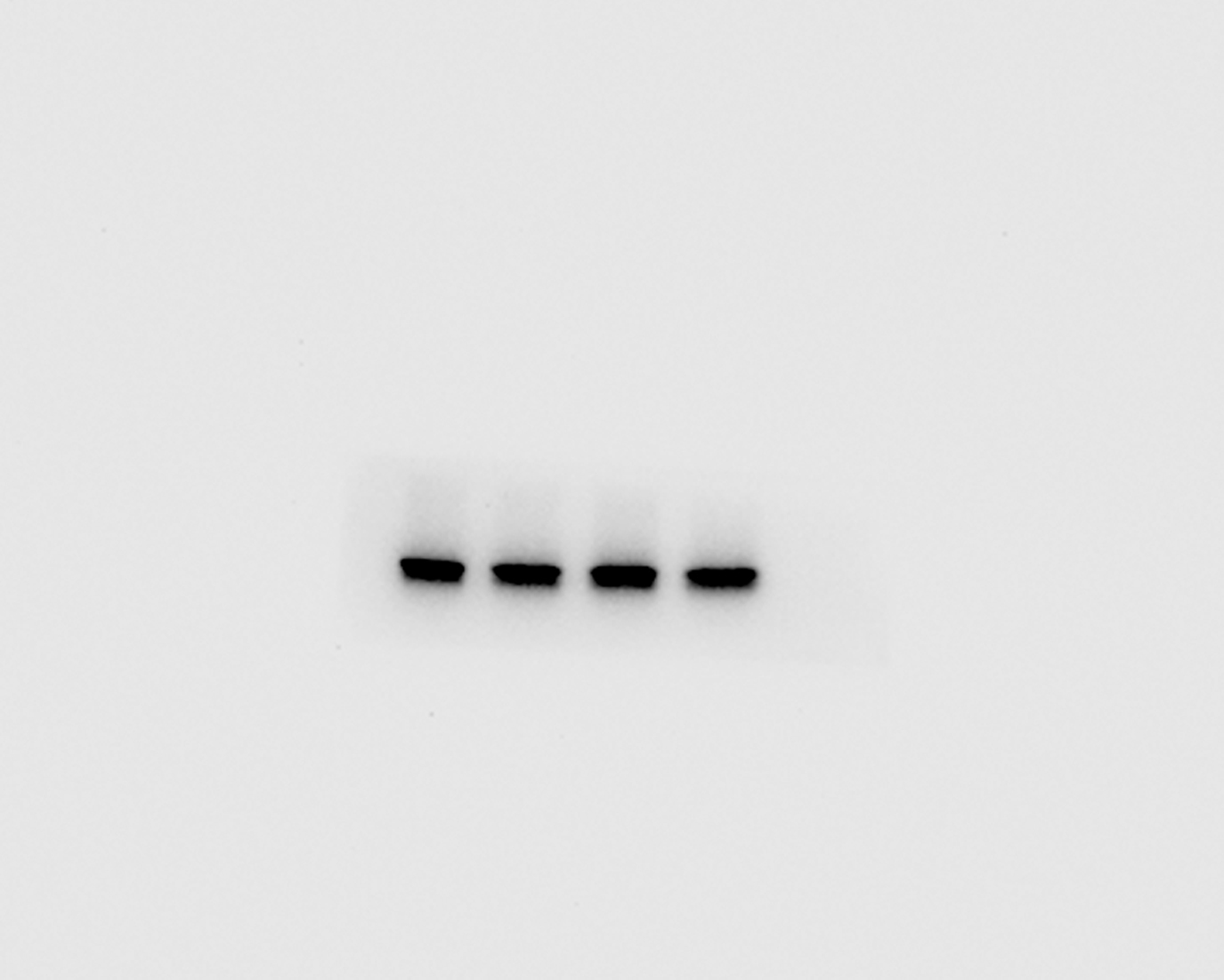

Supplement: Supplementary file 4 — Additional file 4. [file 12917_2022_3533_MOESM4_ESM.jpg]

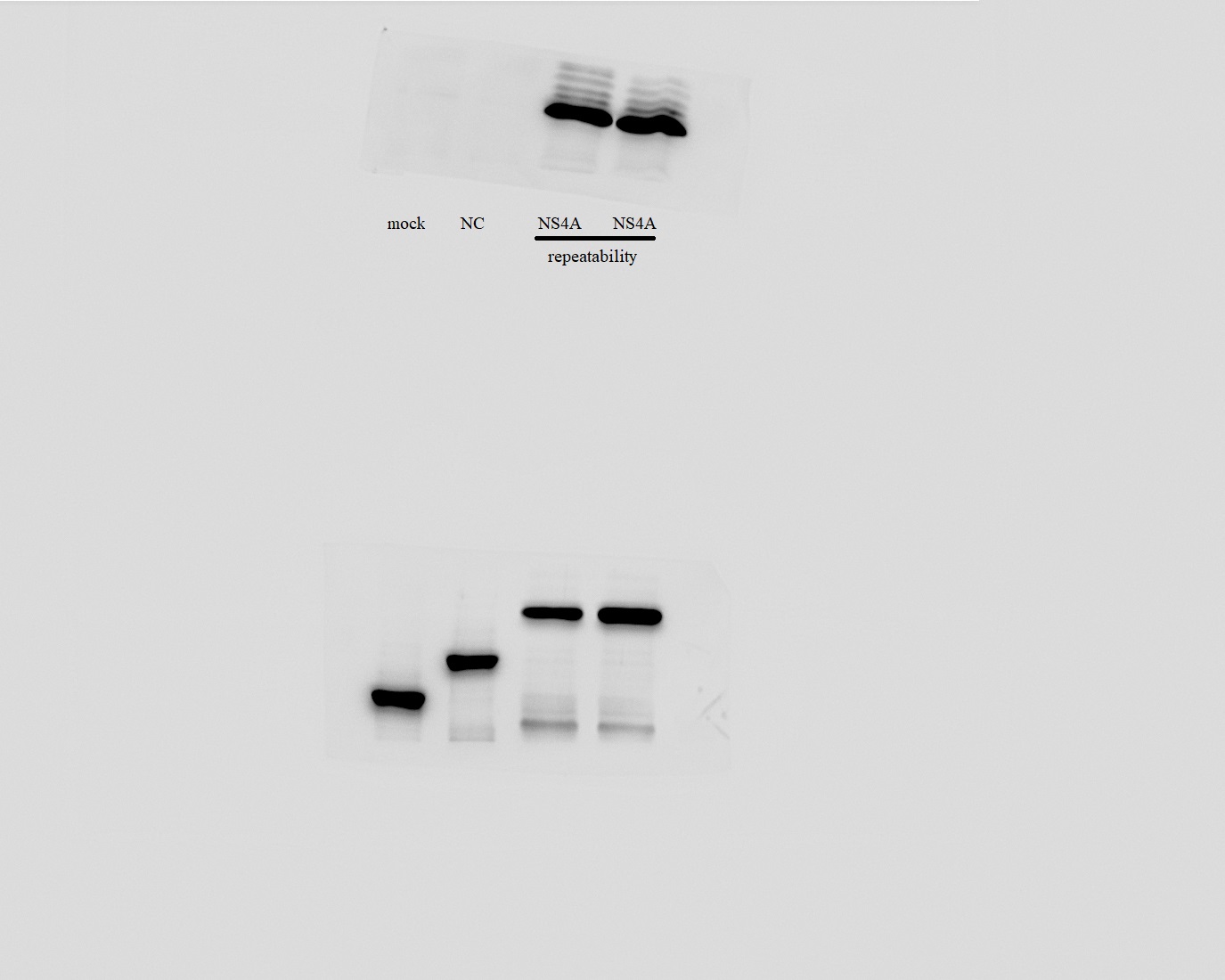

Supplement: Supplementary file 5 — Additional file 5. [file 12917_2022_3533_MOESM5_ESM.jpg]
