# Supplementary material for: Transcriptome analysis of PK-15 cells expressing CSFV NS4A
Source: BMC Vet Res. 2022 Dec 12;18:434. doi: 10.1186/s12917-022-03533-9 (PMC9742017; doi:10.1186/s12917-022-03533-9)
Supplement: Supplementary file 6 — Additional file 6: Supplementary Table S1. Primers used in this study. [file 12917_2022_3533_MOESM6_ESM.docx]

**Supplementary Table S1** Primers used in this study

| **primers** | **Sequence (5′ → 3′)** | **Purpose** |
| --- | --- | --- |
| **CMV-NS4A-Flag-F** | GGAATTCATGTCAACAGCTGAGAATGCCTTG | Amplification of NS4A |
| **CMV-NS4A-Flag-R** | CGGGATCCTCA*CTTATCGTCGTCATCCTTGTAATC*TAGCTCCTTCAATTCTGTCTCC |  |
| **ATF-4-F** | ATACAGGGTGAAGTGGAAATCT | Real-time PCR for detection of ATF-4 |
| **ATF-4-R** | GGAGTCAGGGCTCATACAGAT |  |
| **BAX-F** | ATGATCGCAGCCGTGGACACG | Real-time PCR for detection of BAX |
| **BAX-R** | ACGAAGATGGTCACCGTCTGC |  |
| **p53-F** | GTCGGCTCTGACTGTACCACC | Real-time PCR for detection of p53 |
| **p53-R** | TTCAGCTCCAAGGCGTCATT |  |
| **IL-10-F** | CGGCGCTGTCATCAATTTCTG | Real-time PCR for detection of IL-10 |
| **IL-10-R** | CCCCTCTCTTGGAGCTTGCTA |  |
| **TNF-α-F** | ACCACGCTCTTCTGCCTACTG | Real-time PCR for detection of TNF-α |
| **TNF-α-R** | ACGGGCTTATCTGAGGTTTGA |  |
| **NOD1-F** | ACCGATCCAGTGAGCAGATA | Real-time PCR for detection of NOD1 |
| **NOD1-R** | AAGTCCACCAGCTCCATGAT |  |
| **NLRP3-F** | GAGCCAGAATGGGACAATGCAAAT | Real-time PCR for detection of NLRP3 |
| **NLRP3-R** | CTTTCTTTTTCTTACAAATAGAG |  |
| **TRIM21-F** | CCAGACTCCCCTCTACCCT | Real-time PCR for detection of TRIM21 |
| **TRIM21-R** | TTCCACCGTCATTGAAACC |  |
| **TRIM25-F** | AGGATGTGCGGGTGAGTG | Real-time PCR for detection of TRIM25 |
| **TRIM25-R** | TTGAGGAGGACCTGGTAAATG |  |
| **TRIM40-F** | AAGAGTGCCAGCGATTTATTG | Real-time PCR for detection of TRIM40 |
| **TRIM40-R** | GTGTCAGAGGGTCGGGTG |  |
| **ATG3-F** | CACGACTATGGTTGTTTGGCTATG | Real-time PCR for detection of ATG3 |
| **ATG3-R** | GGTGGAAGGTGAGGGTGATTT |  |
| **CXCL10-F** | CAGTTAGGGCTTGATGTATGGT | Real-time PCR for detection of CXCL10 |
| **CXCL10-R** | GGCACTACTGATAAGGATGGG |  |
| **β-actin-F** | CAAGGACCTCTACGCCAACAC | Real-time PCR for detection of β-actin |
| **β-actin-R** | TGGAGGCGCGATGATCTT |  |

Underlines show restriction enzyme sites, italic show flag tag.
